# Current opinion on guided implant surgery

**DOI:** 10.6026/97320630019786

**Published:** 2023-06-30

**Authors:** Surthi Senthil, Vijayalakshmi R, Burnice Nalina Kumari C, Jaideep Mahendra, Ambalavanan N

**Affiliations:** 1 Department of Periodontology, Meenakshi Ammal Dental College and Hospital, Faculty of Dentistry, Meenakshi Academy of Higher Education and Research, Chennai - 600095, Tamil Nadu

**Keywords:** Guided implant surgery, dental implants, static approach, dynamic approach

## Abstract

Implant dentistry has been evolving with various new technologies, materials and techniques of placement. Conventionally, determination of implant position, size and placement depend on pre-surgical diagnostic imaging, which is limited to two-dimensional radiographs and on the guiding acrylic stent which will be usually prepared over the duplicated casts. Guided implant surgery using cone beam computed tomography, virtual treatment planning software and stereolithographic surgical templates has undoubtedly been a major step toward achieving optimal 3-D implant positioning with respect to both anatomical and prosthetic parameters. This article discusses about the indications, advantages and the concept of guided implant surgery in the successful outcome of the implant placement.

## Background:

Loss of teeth is common due to various reasons such as periodontal disease, poorly maintained and untreated decayed tooth, genetic alteration in enamel and dentin and trauma. [1] Over the years, many ways of treatment for replacement of missing tooth have been developed in various aspects and have been in effect. The dental implant acts as one of the standard treatment modalities for replacement of missing tooth. [2] The current trend in implant surgery is to reduce total rehabilitation duration, by using less invasive surgical techniques with proper treatment planning. [3] The inherent limitations of two-dimensional radiographic techniques led to the adoption of digital computed tomography and subsequently, the more widespread use of dental cone beam computed tomography has allowed the precise three-dimensional evaluation of osseous topography. Digital technology has evolved rapidly and has enabled further improvement in increasing the accuracy. Different concepts of three-dimensional planning, such as computer-guided (static) surgery and computer-navigated (dynamic) surgery, have been proposed and used to transfer digital planning from a personal computer to the surgical field. [3] This review article discusses about the indications, advantages and the concept of guided implant surgery in the successful outcome of the implant placement.

##  Guided implant surgery:

At the end of 1970s, several authors reported on the combined use of stereotaxic frames and computerized tomography scanning of the human head. Later in 1980s, different research groups developed and utilized several software packages to visualize the human head using computerized tomography images. [4] In 1992, a frameless system called the 'Viewing Wand' was the first navigation unit developed surgically by Ontario-based team for neurosurgery. [5] The primary clinical benefits of the Viewing Wand were the significantly improved surgical navigation and clinical safety for the patient during the surgical intervention itself. The surgical paradigm of exposing the tissues to get a better view of the operative area grew outdated and, in some cases, downright reckless, which led to the rise in popularity of surgical navigation. [6] This led to the idea of guided surgery in dentistry with use of various computer softwares. Guided implant surgery allows transferring planned rehabilitation project directly into surgical field. Guided implant surgery protocols are of two types: [3]

[1] Static-guided approach

[2] Dynamic-guided approach

The first system uses a surgical template, obtained from computerized tomographic images. The second approach uses virtual implant positioning from the computerized tomographic images. [7] The treatment protocol for computer assisted implant surgery follows the fundamental steps: [8]

[1] Cone-Beam Computed Tomography (CBCT) scanning

[2] Software program execution

[3] Fabrication of surgical drilling guides in case of static approach

[4] Surgical procedure

## Static guided approach:

Before the surgical procedure, the treatment planning is done and the diagnostic cast is made to determine tooth size, position, contour, and occlusion in the edentulous regions where implants will be inserted. Then, an acrylic vacuum sheet is made to adapt over the diagnostic cast. The occlusal acrylic is removed over the ideal and optional implant sites. Later during surgical procedure, the soft tissue is reflected the template is positioned over teeth and the implant placement is done. In recent years, treatment planning decisions are made using CAD/CAM and can be easily transferred to the surgical treatment phase. [8,9]

## Dynamic guided approach:

Surgical navigation systems can track a surgical tool relative to the patient, and to dynamically position the surgical tool within the patient's pre-surgical computed tomography scan, updated in real time. There are two types of optical motion tracking systems: active and passive. Active tracking system arrays emit infrared light that is tracked to stereo cameras, and passive tracking system arrays use reflective spheres to reflect infrared light emitted from a light source back to a camera.[10] To begin with Scanning of the patient is done and is followed by software planning of the implant position is done. The next step is Image-to-patient registration via registration templates, external registration frames or bone markers. Then the surgery is begun using the navigation of the drill along the predefined surgical plan. [11]

## Advantages of guided implant surgery:

Dynamic navigation can improve the precision of implant placement. [12] When accurate implant angulation is required, which is especially important in the esthetic zone and for screw-retained prosthesis dynamic navigation plays a vital role. It also controls the depth during implant placement such as to avoid damage to nerve or while preparing an osteotomy site adjacent to the sinus floor, when elevating the sinus floor through the implant preparation site. [13]

## Limitations of guided implant surgery:

There might be errors based on the accuracies of materials and types of equipment and techniques used for 3D printing of the surgical templates. Limited accessibility and availability of specific digital service provides another potential problem causing a delay in the treatment. The level of competency of the clinician in handling the guided implant surgery procedure is a crucial factor especially in completely edentulous arches. [14]

An in-vitro study by *Kramer et al*. (2005) compared the accuracy of conventional implant placement and dynamic navigation implant placement methods. The authors concluded that the in-vitro application of a navigation system resulted in an improved precision of insertion surgery regarding the position, angulation and depth of an implant under the experimental conditions and further clinical studies were required to validate this observation. [15]

A systematic review and meta-analysis by *Bover Ramos et al*. (2018) analyzed the accuracy of implant placement using guided implant surgery in experimental models, cadavers and clinical method. It was observed that in terms of horizontal apical deviation and angular deviation, the accuracy of implant placement was lower in cadaver and clinical studies compared to the in-vitro studies. This showed that accuracy of implant placed by guided implant placement can vary clinically than in experimental resin models. [16] A randomized controlled clinical trial by *Nickenig et all*. (2010) evaluated the accuracy of the implant placement using guided surgical method and free-hand conventional method. It was found that there was significantly smaller variation between the planned and the actual implant position done by three-dimensional surgical guide. [17]

Similarly, a randomized controlled trial by *Sondergaard et al*. (2020) compared fully guided with conventionally guided implant surgery performed by dental students in terms of parameters such as facio-lingual crestal deviation, facio-lingual apical deviation, facio-lingual angular deviation, mesio-distal crestal deviation, mesio-distal apical deviation, mesio-distal angular deviation and vertical deviation. Statistically significant differences were seen only in facio-lingual angular deviation and facio-lingual apical deviations which was lowered in fully guided surgery and no significant differences was seen in other parameters. [18]

The accuracy of guided implant surgery and the influence of smoking was first studied by *D'haese et al*. (2012) who had reported that statistically significant differences were found when comparing the accuracy of dental implant placement in smokers to non-smokers which is due to significant thicker supporting mucosal tissues seen in smokers when compared to non-smokers which may explain inaccuracy due to less stability of the scanning prosthesis or the surgical guide. [19]

When the accuracy of implant placement was compared between experienced dental surgeons and inexperienced dental surgeries, *Van de Wiele et al*. (2015) evaluated that only in angulation, the inexperienced group scored lower than that of the experienced surgeons. Hence, the authors came to the conclusion that incorrect guide positioning was the main cause of inaccuracy and surgical experience had played a small role with no major influence on implant placement accuracy. [20] In contrary, *Cassetta et al*. (2017) had reported that the inexperienced group performed better only in the results of angular deviation. He had also added that experienced group showed better accuracy in global apical, coronal deviation. The authors had observed that major difference was seen in the positioning error between experienced and inexperienced group. [21]

In a systematic review by *Kasradze et al*. (2021) influence of various parameters in guided implant surgical procedures was studied. Out of 36 studies analyzed, 35 studies have been reported with deviation at entry point. While when the deviations were analyzed at the apex, out of the 36 studies, 33 were observed with deviations. In implant depth deviation, out of total 36 studies, 22 studies reported the depth deviation. The angular deviations were present in 33 studies out of total 36 studies evaluated. However, with the reported deviations of guided implantation, guided implant placement accuracy was still superior to freehand placement. [22]

On comparing guided flapless surgery with conventional open flap surgery and reporting on patient-centered outcomes three studies were identified in a systematic review done by *Hultin et al*. (2012). [23] These investigations showed that flapless guided surgery resulted in a statistically significant decrease in immediate post-operative discomfort, analgesic use, swelling, oedema, hematoma, bleeding and trismus. Additionally, one of these studies also evaluated guided flapless surgery versus guided open flap surgery and found that the flapless guided technique consistently produced improved outcome measures. These findings are substantiated by the favourable ratings for patient satisfaction and comfort provided by observational studies on patient populations undergoing guided flapless surgery. Thus, with the limited literature the authors reported that flapless guided surgery may have benefits in decreasing patient pain and discomfort in the immediate post-operative period.

A study by *Komiyama et al*. (2008) reported that the duration of the flapless guided immediate implant placement took less than 45 minutes in which the patients reported with minimal pain and discomfort post-operatively. Therefore, the time factor may potentially be a contributing element to the patient's experiences of less pain and discomfort following flapless guided surgery. [24]

## Conclusion:

According to the above mentioned data, guided implant surgery may provide a better accuracy but not a significant difference compared to conventional method of implant placement. Similarly, the variations in the type of guides and the type of technique whether open flap or flapless, also play a role in the position of the implant placement and patient related outcomes. The goal of implant placement is to gain acceptable patient satisfaction with enhanced functional and aesthetic implant prosthesis without complications. Implant surgery has been revolutionized by scientific and technological advancements in digital dentistry and has been well acknowledged as guided implant surgery. As real time and artificial intelligence are well accepted, guided implant surgery might become the futuristic approach for implant placement and will be accepted by the patients enthusiastically if it becomes more affordable and easily accessible.
